# Prognostic value of procalcitonin in hospitalized patients with lower respiratory tract infections

**DOI:** 10.5935/0103-507X.20160019

**Published:** 2016

**Authors:** Vandack Nobre, Isabela Borges

**Affiliations:** 1 Postgraduate Program in Infectious Diseases and Tropical Medicine, Faculdade de Medicina, Universidade Federal de Minas Gerais - Belo Horizonte (MG), Brazil.; 2 Intensive Care Unit, Hospital das Clínicas, Universidade Federal de Minas Gerais - Belo Horizonte (MG), Brazil.

**Keywords:** Infection, Respiratory system, Sepsis, Prognosis, Biomakers, Procalcitonin

## Abstract

Lower respiratory tract infections are common and potentially lethal conditions and are a major cause of inadequate antibiotic prescriptions. Characterization of disease severity and prognostic prediction in affected patients can aid disease management and can increase accuracy in determining the need for and place of hospitalization. The inclusion of biomarkers, particularly procalcitonin, in the decision taken process is a promising strategy. This study aims to present a narrative review of the potential applications and limitations of procalcitonin as a prognostic marker in hospitalized patients with lower respiratory tract infections. The studies on this topic are heterogeneous with respect to procalcitonin measurement techniques, cutoff values, clinical settings, and disease severity. The results show that procalcitonin delivers moderate performance for prognostic prediction in patients with lower respiratory tract infections; its predictive performance was not higher than that of classical methods, and knowledge of procalcitonin levels is most useful when interpreted together with other clinical and laboratory results. Overall, repeated measurement of the procalcitonin levels during the first days of treatment provides more prognostic information than a single measurement; however, information on the cost-effectiveness of this procedure in intensive care patients is lacking. The results of studies that evaluated the prognostic value of initial procalcitonin levels in patients with community-acquired pneumonia are more consistent and have greater potential for practical application; in this case, low procalcitonin levels identify those patients with a low risk of adverse outcomes.

## Abstract (pt)

Infecções do trato respiratório inferior são condições frequentes e potencialmente letais, consistindo nas principais causas de prescrição inadequada de antibióticos. A caracterização de sua gravidade e a predição prognóstica dos pacientes acometidos auxiliam na condução, permitindo maior acerto nas decisões sobre a necessidade e o local de internação, assim como a duração do tratamento. A incorporação de biomarcadores às estratégias classicamente utilizadas representa estratégia promissora, com destaque para a procalcitonina. O objetivo deste artigo foi apresentar uma revisão narrativa sobre a potencial utilidade e as limitações do uso da procalcitonina como um marcador prognóstico em pacientes hospitalizados portadores de infecções do trato respiratório inferior. Os estudos publicados sobre o tema são heterogêneos, no que tange à variedade de técnicas de mensuração da procalcitonina, seus valores de corte, os contextos clínicos e a gravidade dos pacientes incluídos. Os dados obtidos indicam valor moderado da procalcitonina para predizer o prognóstico de pacientes com infecções do trato respiratório inferior, não superior a metodologias classicamente utilizadas, e com utilidade que se faz notar apenas quando interpretados junto a outros dados clínicos e laboratoriais. De modo geral, o comportamento da procalcitonina, ao longo dos primeiros dias de tratamento, fornece mais informações prognósticas do que sua mensuração em um momento isolado, mas faltam informações sobre a custo-efetividade dessa medida em pacientes em terapia intensiva. Estudos que avaliaram o papel prognóstico da procalcitonina inicial em pacientes com pneumonia adquirida na comunidade apresentam resultados mais consistentes e com maior potencial de aplicabilidade prática, mas com utilidade limitada a valores negativos para a seleção de pacientes com baixo risco de evolução desfavorável.

## INTRODUCTION

Lower respiratory tract infections (LRTIs) include a broad and heterogeneous spectrum of diseases that affect patients with varying degrees of severity, from those receiving Primary Care to those receiving intensive care. Moreover, LTRIs are prevalent and potentially lethal. In Brazil, pneumonia is the leading cause of hospitalization in the general population outside obstetric admissions.^(1)^ In European countries, the mortality rates due to community-acquired pneumonia (CAP) range between 1% and 48%, and the costs associated with pneumonia treatment reach 10.1 billion EUR annually.^(2)^ In intensive care units (ICUs), ventilator-associated pneumonia (VAP) is the leading cause of nosocomial infections and death, with mortality rates of 24% - 50%.^(3)^

Therefore, LRTIs are the primary reason for antibiotic prescriptions in outpatient and hospital settings.^(4)^ In ICUs, invasive devices, comorbidities, and previous or acquired immunosuppression all increase patient susceptibility to infection.^(5)^ Moreover, critically ill patients can present atypical clinical and laboratory symptoms, hindering the differential diagnosis of pneumonia and other respiratory conditions. Therefore, antibiotics are excessively and often unnecessarily used, contributing to increased bacterial resistance, cost, and occurrence of side effects.^(6)^

The characterization of disease severity and prognosis in LTRI is another major challenge. Well-validate scores based on clinical, laboratory, and radiological data are routinely applied (Tables 1 and 2) and have a direct impact on patient management. However, these scores may not be sufficient if used alone. For CAP, the most well-validated scoring instruments are the Pneumonia Severity Index (PSI),^(7)^ which has limited applicability owing to its complexity, and the confusion, urea nitrogen, respiratory rate, blood pressure, ≥ 65 years of age (CURB-65),^(8)^ which is simpler and more widely used in clinical practice. A meta-analysis from 2011 assessed the ability of these scores to predict ICU admission and found sensitivities of 74% for PSI values ≥ 4 and 50% for the CURB-65.^(9)^ Therefore, if one uses these scores as the sole tool to define the place of treatment, 26% to 50% of patients will receive a lower level of care than necessary.

**Table 1:** CURB-65 scores for assessment of the severity of community-acquired pneumonia^(8)^

Add 1 point for each variable present:		
New mental confusion Urea > 42mg/dL Respiratory rate ≥ 30irpm SBP < 90mmHg or DBP ≤ 60mmHg Age ≥ 65 years		
**Classification**	**Mortality** **%**	**Recommendation**
0	0.7	Low risk - outpatient treatment
1	3.2	Low risk - outpatient treatment
2	13.0	Intermediate risk - observation
3	17.0	Severe - hospitalization
4	41.5	Severe - hospitalization
5	57.0	Very severe - ICU

SBP - systolic blood pressure; DBP - diastolic blood pressure; ICU - intensive care unit.

**Table 2:** Pneumonia severity index score^(7)^

Consider the total sum of the points:		
Age: Men - age = score Women - age -10 = score Nursing home resident: + 10 Neoplastic disease: + 30 Liver disease: + 20 Congestive heart failure: + 10 Cerebrovascular disease: + 10 Kidney disease: + 10 Altered mental status: + 20 RR > 30bpm: + 20 SBP < 90mmHg: + 20 Axillary temperature < 35 or > 40°C: + 15 HR > 125bpm: + 10 Arterial pH < 7.35: + 30 Urea > 78mg/dL: + 20 Sodium < 130mEq/L: + 20 Glucose > 250mg/dL: + 10 Hematocrit < 30%: + 10 PaO_2_ < 60mmHg: + 10 Pleural effusion: + 10		
**Classification**	**Mortality** **%**	**Recommendation**
I - No points	0.1-0.4	Outpatient treatment
II - < 70	0.6-0.7	Outpatient treatment
III - 70-90	0.9-2.8	Observation
IV - 90-130	8.5-9.3	Hospitalization
V - > 130	27.0-31.0	Hospitalization

RR - respiratory rate; SBP - systolic blood pressure; HR - heart rate; PaO_2_ - partial pressure of arterial oxygen.

Thus, in patients with suspected VAP, severity is typically assessed using the scores more dedicated to critically ill patients, including the Sequential Organ Failure Assessment (SOFA), Acute Physiology and Chronic Health Evaluation (APACHE II), and the Simplified Acute Physiology Score 3 (SAPS 3). However, the diagnosis of VAP is challenging because of the low sensitivity, low specificity, and long processing time of microbiological methods and because of the absence of specific clinical and laboratory signs of VAP.^(3,5)^

In recent years, several research groups have sought to identify blood markers that can help assess the severity and prognosis of LRTI. Combined with classical scores, these markers can be helpful in therapeutic decisions about the place of treatment (i.e., ICU or ward), antibiotic coverage, and length of therapy.^(10)^ One of the most tested biomarkers is procalcitonin (PCT), which has been evaluated in several clinical studies on patients with sepsis.^(10,11)^ Increased serum PCT levels have been associated with the presence, severity, and extent of systemic bacterial infections.^(12,13)^ In addition, PCT increase is dependent on the cytokine cascade and can consequently be quickly neutralized by antibiotics. This characteristic allows PCT kinetics to be used during treatment as a surrogate of the clinical response to treatment and the occurrence of relevant clinical outcomes in patients with sepsis, including the length of hospitalization and mortality. Finally, PCT has been proved to be useful to guide the length of antimicrobial treatment in septic patients.^(11,14)^

The objective of this study is to present a narrative review of the potential benefits and limitations of the use of procalcitonin as a prognostic marker for lower respiratory tract infections in patients in different hospital and clinical settings, with a focus on emergency care and intensive care.

## GENERAL CONSIDERATIONS

Procalcitonin is a 116-amino acid peptide precursor of the hormone calcitonin. In healthy individuals, PCT is secreted only by the neuroendocrine cells of the thyroid. However, during bacterial infections, PCT is released in response to bacterial antigens and to cytokines such as interleukin 1 (IL-1) and tumor necrosis factor alpha (TNF-α) as a secondary mediator of the inflammatory response by many tissues and cell types, including the liver, the kidney, adipocytes, and muscle cells.^(15)^ In viral infections, the production of PCT usually decreases, although it can reach very high levels in severe viral diseases.^(15,16)^ After exposure to endotoxins, the serum levels of PCT begin to increase within 3 hours and reach peak levels between 6 and 24 hours.^(17)^ PCT levels then decrease rapidly when the infection is under control, but they are not affected by the use of anti-inflammatory drugs, including corticosteroids. Increased serum PCT levels can also be observed with non-infectious conditions such as trauma, surgery, pancreatitis, and renal dysfunction. PCT is one of the best-studied biomarkers in clinical practice.^(13,18-21)^

Several studies have evaluated the prognostic role of PCT in patients with sepsis. In a cohort study involving 75 patients, 63 with septic shock and 12 with cardiogenic shock, the PCT levels at the time of inclusion were significantly higher in patients with sepsis; a cutoff value of 6ng/mL distinguished survivors in the ICU from non-survivors in the group of patients with sepsis and yielded a sensitivity of 87.5% and a specificity of 45%.^(22)^ However, the levels of PCT during the first days of antibiotic treatment seem to predict the prognosis more accurately than measurements at any other single time point. In a recently published study, 130 ICU patients with severe sepsis and septic shock were monitored for 18 months.^(23)^ PCT clearance at 24 and 48 hours after the diagnosis of sepsis was significantly higher among survivors, with an area under the curve (AUC) of 0.76 for the prediction of mortality in the ICU, compared with 0.68 for the change in the SOFA score. Charles et al. evaluated other relevant clinical outcomes and reported that the decrease in the levels of PCT between the second and third days of antibiotic treatment was an independent predictor of the response to empirical antimicrobial therapy and was also associated with longer survival.^(24)^ Outside the ICU, the kinetics of PCT as a predictor of mortality in patients with sepsis also achieved positive results. In a cohort study involving 789 patients with severe sepsis or septic shock in an intermediate care unit, PCT levels decreased by less than 15% in 72 hours and by less than 20% between the first 24 and 72 hours; the decreases over these intervals were independent predictors of 30-day mortality, with hazard ratios (HR) of 3.9 (confidence interval - 95%CI, 1.6 - 9.5; p < 0.0001) and 3.1 (95%CI, 1.2 - 7.9; p < 0.001), respectively.^(25)^ The common outcome in the last 3 studies was that most patients had pneumonia as the infection source, at frequencies of 44%, 52%, and 51%, respectively.

## PROGNOSTIC VALUE OF PROCALCITONIN IN LOWER RESPIRATORY TRACT INFECTIONS

### Patients with lower respiratory tract infections treated in emergency care services

The main studies that have evaluated the prognostic value of PCT in patients receiving emergency care are summarized in table 3. To assess the usefulness of PCT in patients with dyspnea in the emergency room, Italian and American researchers investigated 2 cohorts involving a total of 453 patients.^(26)^ Circulating levels of PCT were measured upon admission, and were then associated with the final diagnosis and with 90-day and 1-year mortality rates. Among the patients studied, 60 patients had an initial diagnosis of pneumonia, among whom 30 had decompensated heart failure as the underlying condition. Considering all of the patients, serum PCT levels were higher in the patients who died at 90 days (0.13 [0.08 - 0.41] ng/mL versus 0.06 [0.04 - 0.10] ng/mL; p < 0.001) or at 1 year (0.12 [0.08 - 0.38] ng/mL versus 0.06 [0.04 - 0.10] ng/mL p < 0.001) compared with survivors. Multivariate analysis indicated that serum PCT levels were independently associated with 1-year mortality (HR = 1.8, 95%CI, 1.4 - 2.3; p < 0.001); however, there was no significant association in the group of patients with a specific diagnosis of respiratory infection.

**Table 3:** Studies that evaluated the role of procalcitonin in patients with lower respiratory tract infections in emergency care units

**Author**	**Year**	**Country**	**Study design**	**Sample size (N)**	**Inclusion criteria**	**Outcomes**	**Results**	**Observations**
Alba et al.^(26)^	2015	United States Italy	Bicenter, prospective cohort	453	Dyspnea	Primary: diagnosis of pneumonia Secondary: one-year mortality	Primary outcome: AUC 0.84 for PCT > 0.1 ng/mL Secondary outcome: PCT as an independent predictor of one-year mortality (HR: 1.8; 95%CI: 1.4-2.3). No correlation in patients with pneumonia	Prognostic value as secondary outcome
Travaglino et al.^(27)^	2012	Italy	Multicenter, prospective cohort	128	Fever	Levels of MR-proADM and PCT compared with APACHE II	Positive correlation between APACHE II scores and MR-proADM (r = 0.66) and between MR-proADM and PCT (r = 0.54). No correlation between PCT and APACHE II	Small sample size, LRTI as subgroup
Ugajin et al.^(28)^	2014	Japan	Single-center, retrospective	213	CAP	28-day mortality and need for intensive care	No correlation with mortality. PCT ≥ 10ng/mL was associated with a higher probability of need for intensive care	Semiquantitative measurement
Huang et al.^(29)^	2008	United States	Multicenter, prospective cohort	1,651	CAP	30-day mortality	Low PCT values (< 0.1ng/mL) were associated with lower mortality in high-risk patients (Negative LR of 0.09)	Large sample size. Wide confidence interval
Krüger et al.^(30)^	2008	Germany	Multicenter, prospective cohort	1,671	CAP	28-day mortality Correlation with CRP, WBC, and CURB-65	Accuracy similar to that of CURB-65. Positive correlation with 28-day mortality. Identification of patients with low risk of death	Large sample size but few high-risk patients
Schuetz et al.^(31)^	2011	Switzerland	Multicenter, retrospective	925	CAP	30-day mortality Adverse events in 30 days	Initial levels were weak predictors of mortality (AUC: 0.6). Sequential levels were more useful in predicting adverse events	Large sample size, sequential measurements
Ramirez et al.^(32)^	2011	Spain	Bicenter, prospective cohort	685	CAP	Admission to intensive care	PCT < 0.35ng/dL safely ensured the lack of need of intensive care for severe CAP	Patients with higher severity criteria were excluded
Kutz et al.^(33)^	2015	Switzerland	Systematic review and meta-analysis	2,065	CAP	30-day mortality Treatment failure	PCT < 0.25ng/dL with VPN of 89.2% for treatment failure and 97.5% for mortality	Secondary analysis of clinical trials. Without observational data
Park et al.^(34)^	2012	South Korea	Single-center, prospective	126	CAP	28-day mortality	AUC of 0.82. Addition of prediction to classical scores	Small sample size

AUC - area under the curve; PCT - procalcitonin; HR - hazard ratio; MR-proADM - pro-adrenomedullin; APACHE II - Acute Physiology and Chronic Health evaluation II; LRTI - lower respiratory tract infections; LR - likelihood ratio; CAP - community-acquired pneumonia; NPV - negative predictive value.

In the assessment of febrile patients admitted to an emergency care unit, a multicenter observational study evaluated 128 patients with suspected severe infections, including LRTI, and assessed the prognostic value of PCT and pro-adrenomedullin (MR-proADM) upon admission, in comparison with APACHE II scores, as indirect indicators of mortality.^(27)^ Of the total sample, 44 patients had LRTI, and most of these cases involved pneumonia (n = 31). In patients with LRTI, moderate positive correlations were found between APACHE II scores and MR-proADM (R = 0.66, p = 0.0002) and between MR-proADM and PCT (R = 0.54, p = 0.0001). There was no significant correlation between serum PCT levels and APACHE II scores.

Measuring PCT levels using a semiquantitative method, such as an immunochromatographic assay, is unsatisfactory. The prognostic value (28-day mortality and need for ICU admission) of such a method was assessed in a retrospective study involving 213 patients who were admitted to an emergency care unit with a diagnosis of CAP.^(28)^ There was no significant difference in mortality based on the observed PCT levels. Only levels > 10ng/mL (which limits the sensitivity and negative predictive value (NPV) of the marker) were significantly associated with a need for ICU admission (p < 0.001); however, the performance of PCT as a prognostic marker was lower than that of classical scores and other laboratory markers (AUC of 0.87 for PSI, 0.86 for CUEB-65, 0.85 for the urea/albumin ratio, and 0.82 for semiquantitative PCT; p < 0.001).

A multicenter cohort study conducted in 28 emergency care units in the US evaluated the prognostic value of PCT using a larger cohort of 1,651 patients with CAP.^(29)^ In most cases, PCT levels upon admission did not provide any prognostic information. Only PCT values < 0.1ng/mL in high-risk patients (PSI IV and V) were significantly associated with lower 30-day mortality, with a negative likelihood ratio of 0.09 in this group of patients.

In 2008, a German group evaluated the prognostic value of initial PCT levels in 1,671 patients who received emergency care with a diagnosis of CAP.^(30)^ In that study, PCT levels were significantly higher in severely ill patients, as assessed by CURB-65 score. The highest levels of PCT were observed in 70 patients who died during the 28-day follow-up period (0.88 [0.32 - 3.38] ng/mL versus 0.13 [0.08 - 0.38] ng/mL; p = 0.0001). In the ROC curve, the AUC for PCT was 0.80 (0.75 - 0.84), which was similar to that of the CURB-65 score. These results were encouraging but were not reproduced in a validation cohort study (ProHOSP) involving 925 patients with CAP.^(31)^ In this case, the initial serum levels of PCT showed only moderate performance in predicting 30-day mortality, with an AUC of 0.6. Sequential measurements of PCT resulted in a slight improvement in performance, with AUCs of 0.61, 0.68, and 0.73 on days 3, 5, and 7, respectively. However, the prognostic value of PCT was not superior to the scores traditionally used for prediction of mortality (CURB-65 and PSI).

Another study evaluated the usefulness of initial PCT levels in predicting ICU admission included 685 patients with CAP who were treated in emergency care units; it indicated that low serum levels of PCT (< 0.35ng/mL) were associated with a decreased severity of infections and ensured the safe management of patients outside the ICU.^(32)^

A systematic review evaluated PCT values upon admission in patients with LRTI in emergency units and showed that lower serum levels of PCT were significantly associated with treatment failure (AUC 0.64, 95%CI, 0.61 - 0,67; OR 1.85, 95%CI, 1.61 - 2.12; p < 0.0001) and with 30-day mortality (AUC 0.67, 95%CI, 0.63 - 0.71; OR 1.82, 95%CI, 1.45 - 2.29; p < 0.001), although the correlation was only poor to moderate. A cutoff value of 0.25ng/mL had an NPV of 89.2% and 97.5% and a sensitivity of 65.6% and 72.5% for treatment failure and mortality, respectively, and reached statistical significance.^(33)^

The most satisfactory results in an emergency care setting were presented by a South Korean study published in 2012.^(34)^ The authors evaluated the ability of PCT to predict 28-day mortality in 126 patients admitted to an emergency care unit with clinical and radiological signs of CAP. The average serum level of PCT in patients with PSI I and II was 0.1ng/mL, compared with 0.61ng/mL in patients with PSI V. Patients with CURB-65 scores of 0 or 1 had a mean PCT level of 0.19ng/mL, compared with 4.75ng/mL in those with a score ≥ 3. The average PCT levels upon admission were significantly higher in non-survivors than in survivors (1.96ng/mL versus 0.18ng/mL, p < 0.01) and showed an AUC of 0.82, which was higher than that of the other biomarkers studied (C-reactive protein [CRP], erythrocyte sedimentation rate, and white blood cells) but was similar to that of classical scores (PSI, 0.87; CURB-65 score, 0.86; Infectious Diseases Society of America/American Thoracic Society [IDSA/ATS] score, 0.84). Furthermore, the addition of PCT to the scores significantly increased their predictive accuracy for 28-day mortality.

### Hospitalized patients diagnosed with community-acquired pneumonia

Table 4 summarizes the main studies that have evaluated the prognostic value of PCT in hospitalized patients with CAP. The clinical profile of the patients included in these studies was similar to that of patients evaluated in the studies above, i.e., they were initially evaluated in emergency care units, although the measured outcomes may have been different. When examining the use of PCT for the prognostic prediction of hospitalized patients with respiratory infections, a cohort study from 2014 involving 101 hospitalized patients with CAP found results similar to those of the aforementioned studies; it showed that PCT had low to moderate accuracy for predicting 30-day mortality in this group of patients, with an AUC value of 0.66 (95%CI, 0.54 - 0.78; p ≤ 0.012) The best cutoff value found, 2.56ng/mL, presented a sensitivity of 76% and a specificity of 61.8% for predicting mortality.^(35)^ Another prospective study involving 170 hospitalized patients with CAP showed the superior performance of initial PCT levels in predicting survival at 30 days (AUC for mortality, 0.8; 95%CI, 0.7 - 0.9), although it used a semiquantitative method to measure PCT. The accuracy of PCT was higher than that of other markers, including CRP; however, it was less accurate than the CURB-65 (AUC 0.88) and PSI (AUC 0.89) scores.^(36)^ Furthermore, in a study that evaluated the ability of PCT and other markers to predict treatment failure (occurrence of septic shock, need for mechanical ventilation, or death within 72 hours) and included 453 patients with a diagnosis of CAP, higher levels of PCT (averaging 3.36ng/mL) measured at the baseline the were independent predictors of early treatment failure, with low levels presenting a high NPV for this outcome (0.95).^(37)^

**Table 4:** Studies that evaluated the role of procalcitonin in hospitalized patients with community-acquired pneumonia

**Author**	**Year**	**Country**	**Study design**	**Sample size (N)**	**Inclusion criteria**	**Outcomes**	**Results**	**Observations**
Andrijevic et al.^(35)^	2014	Serbia	Single-center, prospective cohort	101	CAP	30-day mortality	Weak predictor of mortality (AUC: 0.66). PCT > 2.56ng/mL with sensitivity of 76% and specificity of 61.8%	Small sample size
Kasamatsu et al.^(36)^	2012	Japan	Single-center, prospective cohort	170	CAP	30-day mortalitys	AUC of 0.8. Correlation with PSI (0.56) and CURB-65 (0.58)	Semiquantitative measurement
Menéndez et al.^(37)^	2008	Spain	Bicenter, prospective cohort	453	CAP	Treatment failure (septic shock, mechanical ventilation, or death)	High PCT on day 1 as good predictor of early failure (OR of 2.7). Decreased levels had a strong VPN (0.95)	Cutoff values were not defined

CAP - community-acquired pneumonia; AUC - area under the curve; PCT - procalcitonin; PSI - Pneumonia Severity Index; OR - odds ratio; NPV - negative predictive value.

### Patients with pneumonia admitted to an intensive care unit

The prognostic evaluation of severely ill patients is complex because of the multiplicity of variables that influence their outcomes. Studies on PCT in patients with respiratory infections in intensive care indicated promising but conflicting results (Table 5).

**Table 5:** Studies that evaluated the role of procalcitonin in patients with pneumonia admitted to the intensive care unit

**Author**	**Year**	**Country**	**Study design**	**Sample size (N)**	**Inclusion criteria**	**Outcomes**	**Results**	**Observations**
Shi et al.^(38)^	2014	China	Single-center, prospective cohort	60	Nosocomial pneumonia	Clinical efficacy and microbiological response	No correlations with absolute PCT values. PCT kinetics was the best single indicator of clinical efficacy (AUC: 0.79)	Older people
Boussekey et al.^(39)^	2006	France	Single-center, prospective cohort	100	Severe CAP	Mortality in the ICU	Increased PCT levels on days 1 to 3 were associated with mortality (OR: 4.539)	Semiquantitative measurement
Rammaert et al.^(40)^	2009	France	Single-center, prospective cohort	116	Exacerbated COPD	Mortality in the ICU	PCT was independently associated with mortality (HR 1.01; 1.00 - 1.03)	Only patients who underwent invasive ventilation were included
Bloos et al.^(41)^	2011	Canada United States Europe	Multicenter, prospective cohort	175	CAP and nosocomial pneumonia, including VAP	28-day mortality	AUC of 0.70 and 0.74 as initial and maximum PCT levels. Cutoff PCT values of 1.1ng/mL (OR, 7.0; 95%CI, 2.6 - 25.2) and 7.8ng/mL (OR, 5.7; 95%CI, 2.5 -13.1), respectively	Semiquantitative measurement. Wide confidence interval for cutoff values
Kutz et al.^(33)^	2015	Switzerland	Systematic review and meta-analysis	598	CAP, nosocomial pneumonia, including VAP and other	30-day mortality. Treatment failure	No correlation found. AUC of 0.50 (95%CI, 0.44 - 0.56) OR of 1.05 (95%CI, 0.81 - 1.37)	Secondary analysis of clinical trials. Without observational data
Luyt et al.^(42)^	2005	France	Single-center, prospective cohort	63	VAP	Combined outcome: 28-day mortality, VAP recurrence, or extrapulmonary infection	PCT levels on days 1, 3 and 7 were strong predictors of poor outcome (OR: 12.3 on day 1 and 64.22 on day 7)	Small sample size. High incidence rate for the outcome
Seligman et al.^(43)^	2006	Brazil	Single-center, prospective cohort	71	VAP	28-day mortality	PCT kinetics was an independent associated factor (OR, 4.43; 95%CI, 43.44 - 59.03)	Small sample size and wide confidence interval
Hillas et al.^(44)^	2010	Greece	Single-center, prospective cohort	45	VAP	28-day mortality and septic shock	AUC for mortality on day 1 of 0.79 (0.66 - 0.92) and 0.88 on day 7 (0.77 - 0.99). No correlation in the multivariate analysis	Small sample size
Boeck et al.^(45)^	2011	United States Switzerland	Multicenter, prospective cohort	101	VAP	28-day mortality	PCT on admission was higher among non-survivors (1.36 versus 0.58ng/mL; p = 0.017)	Secondary outcome
Tanriverdi et al.^(46)^	2015	Turkey	Single-center, prospective cohort	45	VAP	28-day mortality	PCT > 1ng/mL on day 3 was the strongest predictor (OR, 5.95; 95%CI, 1.58 - 22.32)	Small sample size. Wide confidence interval

PCT - procalcitonin; AUC - area under the curve; CAP - community-acquired pneumonia; OR - odds ratio; COPD - chronic obstructive pulmonary disease; HR - hazard ratio; VAP - ventilator-associated pneumonia; 95%CI - 95% confidence interval.

In a recent study involving 60 older patients with nosocomial pneumonia who were admitted to the ICU, the evaluation of PCT kinetics between the time of admission and the third day of follow-up was the best single predictor of therapeutic efficacy, with an AUC of 0.79 (p < 0.001).^(38)^ Similarly, a study from 2006 evaluated the prognostic value of PCT kinetics in patients with CAP who were admitted to the ICU; the study indicated favorable results.^(39)^ One hundred patients were included, and serum PCT levels were measured on days 1 and 3. An increase in PCT levels between days 1 and 3 was independently associated with mortality in the ICU, with an OR of 4.539 (95%CI, 1.31 - 15.75; p ≤ 0.017). In intubated patients, PCT levels < 0.95ng/mL on day 3 were associated with favorable outcomes, with a survival rate of 95% in the ICU.

The prognostic accuracy of PCT was also evaluated in patients with exacerbated chronic obstructive pulmonary disease who required mechanical ventilation. A study from 2009 involving 116 patients demonstrated that PCT levels upon ICU admission were independently but only slightly associated with mortality in intensive care (HR of 1.01; 95%CI, 1.00 - 1.03; p = 0.018),^(40)^ Mortality was significantly higher in patients with PCT > 0.24ng/mL compared with those with PCT < 0.24ng/mL (36% versus 17%; p = 0.031; OR, 2.7; 95%CI, 1.10 - 6.50)

Among the studies that evaluated patients with VAP, Bloos et al. (2011) conducted a multicenter study and determined PCT levels in 175 critically ill patients on mechanical ventilation, of whom 57 presented CAP, 57 presented nosocomial pneumonia, and 61 presented VAP.^(41)^ Notably, the initial PCT levels were higher in patients with CAP than in those with VAP, with a median of 2.4 (0.95 - 15.8) versus 0.7 (0.30-2.15) ng/mL (p < 0.001). The initial and maximal levels of PCT were correlated with the maximal SOFA scores. The AUC values for 28-day mortality were slightly higher for the maximum PCT (0.74) than for the initial PCT (0.70) and APACHE II scores (0.69). The optimal cutoff for the maximal PCT level to predict 28-day mortality was 7.8ng/mL (OR, 5.7; 95%CI, 4.0 - 18.7). However, these findings were not confirmed by a recently published meta-analysis involving 14 studies and 598 patients in the ICU, in which initial PCT levels were not associated with treatment failure or death.^(33)^

In 2005, a prospective study evaluated 63 patients and measured PCT on days 1, 3 and 7 of follow-up to assess PCT kinetics during VAP.^(42)^ A cutoff value of 1ng/mL on day 1 had a sensitivity of 83% and a specificity of 64% in predicting the occurrence of an unfavorable outcome (28-day mortality, VAP recurrence, or extrapulmonary infection). On day 3, a cutoff value of 1.5ng/mL had a sensitivity of 74% and a specificity of 84%, while on day 7, a cutoff of 0.5ng/mL had a sensitivity of 90% and a specificity of 88% for the same outcome. In a multivariate analysis, PCT values higher than those reported on each of the assessment days were independently associated with the above-mentioned unfavorable outcomes, with OR values of 12.3 on day 1 and 64.2 on day 7.

In Brazil, a study evaluated the prognostic value of PCT, CRP, clinical pulmonary infection score (CPIS), and SOFA upon admission and at day 4 of follow-up in 71 patients with VAP.^(43)^ Survival at 28 days was strongly associated with a decrease in the PCT levels between the evaluation days, with an OR of 5.67, which was higher than that obtained for the decreases in CRP and SOFA scores (ORs of 3.78 and 3.08, respectively). In the analysis, only the decreases in PCT and CRP were independent risk factors for predicting survival. Similarly, a Greek study from 2010 evaluated the performance of PCT and CRP in predicting progression to septic shock and 28-day mortality in a prospective cohort of 45 patients with VAP, measuring biomarkers on days 1, 4, and 7 after admission.^(44)^ The AUC values for predicting survival using PCT on days 1 and 7 were 0.79 (95%CI, 0.66 - 0.92) and 0.88 (95%CI, 0.77 - 0.99), respectively. However, in the multivariate analysis, variations in the PCT levels between days 1 and 7 (OR, 7.23; 95%CI, 0.00 - 0.468) and in CRP between days 4 and 7 (OR, 4.59; 95%CI, 0.013 - 0.824) were not significant predictors of mortality. No single level of these biomarkers was associated with the studied outcomes.

In another cohort, involving 101 patients with a diagnosis of VAP significantly increased PCT levels upon admission were observed among non-survivors as compared to survivors (1.36ng/mL versus 0.58ng/mL; p = 0.017).^(45)^ The average relative decrease in PCT levels 72 hours after the onset of symptoms was 26% in survivors and 7% in non-survivors. A recent prospective cohort study involving 45 patients evaluated the association between 28-day mortality and the kinetics of PCT or of CRP between admission and days 3 and 7 of follow-up,^(46)^ and it found no difference in PCT levels upon admission between survivors and non-survivors; however, the PCT levels on days 3 and 7 were significantly higher among non-survivors. Moreover, PCT levels decreased significantly between day 0 and day 7 among survivors, whereas CRP levels did not change. On day 3, PCT levels > 1ng/mL proved to be strong predictors of death, with an OR of 5.95 (1.58 - 22.32).

### Limitations of the studies on the subject

Although many studies have evaluated the prognostic value of PCT in patients with LRTI, it seems difficult to establish the real utility of PCT in this context; the studies are very heterogeneous, which limits aggregated data analysis and comparisons among results. The heterogeneity arises from the fact that these studies used several diagnostic methods and distinct diagnostic criteria for LRTI, and were conducted in different settings (emergency units, ICUs, and wards). Most studies examined a small sample size and experienced a high number of losses after initial screening, which limit the power of statistical inferences and the validity of the results presented. In addition, they used distinct methods of PCT measurement, including semiquantitative techniques, and the evaluated patients presented with different levels of disease severity. Other relevant limitations include a lack of patient follow-up beyond 28 days in most studies, a lack of adjustment for potential confounders (including renal failure),^(47)^ a lack of cost-effectiveness analysis, and a lack of testing in validation cohorts. Furthermore, few experimental studies have evaluated the role of PCT measurement as a strategy for the clinical management of patients with LRTI.

## DISCUSSION

Most of the relevant studies have concluded that serum PCT levels may be useful for predicting the prognosis of patients with LRTI but do not perform better than classical laboratory methods and clinical scores. Several studies evaluated the serum levels of PCT in patients with CAP in emergency units and used large sample sizes. Some of these studies found that serum levels of PCT were independent predictors of death,^(29-31,33,34)^ but also found that the performance of PCT neither exceeded that of well-validated scores, such as CURB-65 and PSI, nor added new information that aided in the evaluation of patients with CAP. Therefore, in this scenario, PCT should be regarded as an additional parameter that increases the accuracy of classical methods but has limited value when used alone. Moreover, the use of PCT seems most useful when low PCT levels are detected, enabling the identification of patients with a lower risk of adverse outcomes. However, optimal cutoff values for such identification have not been established.

In the evaluation of hospitalized patients or patients with nosocomial pneumonia (including VAP) or CAP, the results are less robust, likely because of the small sample sizes and the smaller number of studies. Still, among such patients, the circulating levels of PCT during the first days of antimicrobial treatment seem more informative and accurate than the use of separate measurements at the beginning of treatment, although analyses of the cost-effectiveness of this approach have not been done. In urgent care, we should consider PCT as an additional parameter that is available for the overall clinical assessment of patients. Moreover, in these scenarios, the VPN of PCT for mortality and other negative outcomes proved to be higher, and low serum levels were better predictors of prognosis in different groups of patients. In terms of practical applicability, low levels of PCT in patients without an obvious indication for intensive care would increase confidence in the decision to maintain them outside of the ICU.

For PCT to become effective for routine use as an auxiliary marker for prognostic prediction in patients with LRTI, intervention studies must be conducted to experimentally evaluate the effect of PCT on relevant outcomes in patients with LRTI. An evaluation of this type was the multicenter study conducted in Denmark by Jensen et al.,^(48)^ who tested the usefulness of performing daily PCT measurements as an indicator for the need to increase therapeutic (including antimicrobial spectrum escalation) and diagnostic interventions (eg, computed tomography) related to infectious complications in critically ill patients. The PCT was measured daily in patients from the experimental group, and the "alert procalcitonin" was defined as (1) PCT > 1.0ng/mL without decreasing by at least 10% from the previous day or (2) an isolated PCT level > 1.0ng/mL upon admission. In these cases, diagnositc and therapeutic interventions were intensified. The authors found no difference between the outcomes observed for the control group and for the group managed using PCT, which included 28-day mortality, duration of organ dysfunction, and length of ICU stay.

## CONCLUSION

The use of procalcitonin as a prognostic marker in patients with lower respiratory tract infections has limited practical applicability and, when used alone, does not perform better than other methods that are typically used for this purpose in different hospital settings.

## References

[1] Brasil, Ministério da Saúde, DATASUS Morbidade hospitalar do SUS.

[2] Welte T, Torres A, Nathwani D (2012). Clinical and economic burden of community-acquired pneumonia
among adults in Europe. Thorax.

[3] Chastre J, Fagon JY (2002). Ventilator-associated pneumonia. Am J Respir Crit Care Med.

[4] Mizgerd JP (2008). Acute lower respiratory tract infection. N Engl J Med.

[5] Bonten MJ (2011). Healthcare epidemiology: Ventilator-associated pneumonia:
preventing the inevitable. Clin Infect Dis.

[6] Wenzel RP (2004). The antibiotic pipeline-challenges, costs, and
values. N Engl J Med.

[7] Fine MJ, Auble TE, Yealy DM, Hanusa BH, Weissfeld LA, Singer DE, Coley CM, Marrie TJ, Kapoor WN (1997). A prediction rule to identify low-risk patients with
community-acquired pneumonia. N Engl J Med.

[8] Lim WS, van der Eerden MM, Laing R, Boersma WG, Karalus N, Town GI (2003). Defining community acquired pneumonia severity on presentation to
hospital: an international derivation and validation study. Thorax.

[9] Chalmers JD, Mandal P, Singanayagam A, Akram AR, Choudhury G, Short PM (2011). Severity assessment tools to guide ICU admission in
community-acquired pneumonia: systematic review and
meta-analysis. Intensive Care Med.

[10] Prkno A, Wacker C, Brunkhorst FM, Schlattmann P (2013). Procalcitonin-guided therapy in intensive care unit patients with
severe sepsis and septic shock-a systematic review and
meta-analysis. Crit Care.

[11] Schuetz P, Amin DN, Greenwald JL (2012). Role of procalcitonin in managing adult patients with respiratory
tract infections. Chest.

[12] Uzzan B, Cohen R, Nicolas P, Cucherat M, Perret GY (2006). Procalcitonin as a diagnostic test for sepsis in critically ill
adults and after surgery or trauma: a systematic review and
meta-analysis. Crit Care Med.

[13] Wacker C, Prkno A, Brunkhorst FM, Schlattmann P (2013). Procalcitonin as a diagnostic marker for sepsis: a systematic
review and meta-analysis. Lancet Infect Dis.

[14] Charles PE, Tinel C, Barbar S, Aho S, Prin S, Doise JM (2009). Procalcitonin kinetics within the first days of sepsis:
relationship with the appropriateness of antibiotic therapy and the
outcome. Crit Care.

[15] Müller B, White JC, Nylén ES, Snider RH, Becker KL, Habener JF (2001). Ubiquitous expression of the calcitonin-I gene in multiple
tissues in response to sepsis. J Clin Endocrinol Metab.

[16] Paiva MB, Botoni FA, Teixeira AL, Miranda AS, Oliveira CR, Abrahão Jde O (2012). The behavior and diagnostic utility of procalcitonin and five
other inflammatory molecules in critically ill patients with respiratory
distress and suspected 2009 influenza a H1N1 infection. Clinics (Sao Paulo).

[17] Dantona P, Nix D, Wilson MF, Aljada A, Love J, Assiscot M (1994). Procalcitonin increase after endotoxin injection in normal
subjects. J Clin Endocrinol Metab.

[18] Müller B, Harbarth S, Stolz D, Bingisser R, Mueller C, Leuppi J (2007). Diagnostic and prognostic accuracy of clinical and laboratory
parameters in community-acquired pneumonia. BMC Infect Dis.

[19] Nobre V, Harbarth S, Graf JD, Rohner P, Pugin J (2008). Use of procalcitonin to shorten antibiotic treatment duration in
septic patients: a randomized trial. Am J Respir Crit Care Med.

[20] Bouadma L, Luyt CE, Tubach F, Cracco C, Alvarez A, Schwebel C, Schortgen F, Lasocki S, Veber B, Dehoux M, Bernard M, Pasquet B, Régnier B, Brun-Buisson C, Chastre J, Wolff M, PRORATA trial group (2010). Use of procalcitonin to reduce patients' exposure to antibiotics
in intensive care units (PRORATA trial): a multicentre randomised controlled
trial. Lancet.

[21] Schuetz P, Müller B, Christ-Crain M, Stolz D, Tamm M, Bouadma L (2013). Procalcitonin to initiate or discontinue antibiotics in acute
respiratory tract infections. Evid Based Child Health.

[22] Clec'h C, Ferriere F, Karoubi P, Fosse JP, Cupa M, Hoang P (2004). Diagnostic and prognostic value of procalcitonin in patients with
septic shock. Crit Care Med.

[23] de Azevedo JR, Torres OJ, Beraldi RA, Ribas CA, Malafaia O (2015). Prognostic evaluation of severe sepsis and septic shock:
procalcitonin clearance? Sequential Organ Failure Assessment. J Crit Care.

[24] Charles PE, Tinel C, Barbar S, Aho S, Prin S, Doise JM (2009). Procalcitonin kinetics within the first days of sepsis:
relationship with the appropriateness of antibiotic therapy and the
outcome. Crit Care.

[25] Pieralli F, Vannucchi V, Mancini A, Antonielli E, Luise F, Sammicheli L (2015). Procalcitonin kinetics in the first 72 hours predicts 30-day
mortality in severely ill septic patients admitted to an intermediate care
unit. J Clin Med Res.

[26] Alba GA, Truong QA, Gaggin HK, Gandhi PU, De Berardinis B, Magrini L, Bajwa EK, Di Somma S, Januzzi JL, Global Research on Acute Conditions Team (GREAT) Network (2016). Diagnostic and Prognostic utility of procalcitonin in patients
presenting to the emergency department with dyspnea. Am J Med.

[27] Travaglino F, De Berardinis B, Magrini L, Bongiovanni C, Candelli M, Silveri NG, et al (2012). S. Utility of Procalcitonin (PCT) and Mid regional
pro-Adrenomedullin (MR-proADM) in risk stratification of critically ill
febrile patients in Emergency Department (ED). A comparison with APACHE II
score. BMC Infect Dis.

[28] Ugajin M, Yamaki K, Hirasawa N, Yagi T (2014). Predictive values of semi-quantitative procalcitonin test and
common biomarkers for the clinical outcomes of community-acquired
pneumonia. Respir Care.

[29] Huang DT, Weissfeld LA, Kellum JA, Yealy DM, Kong L, Martino M, Angus DC, GenIMS Investigators (2008). Risk prediction with procalcitonin and clinical rules in
community-acquired pneumonia. Ann Emerg Med.

[30] Krüger S, Ewig S, Marre R, Papassotiriou J, Richter K, von Baum H, Suttorp N, Welte T, CAPNETZ Study Group (2008). Procalcitonin predicts patients at low risk of death from
community-acquired pneumonia across all CRB-65 classes. Eur Respir J.

[31] Schuetz P, Suter-Widmer I, Chaudri A, Christ-Crain M, Zimmerli W, Mueller B, Procalcitonin-Guided Antibiotic Therapy and Hospitalisation in
Patients with Lower Respiratory Tract Infections Study Group (2011). Prognostic value of procalcitonin in community-acquired
pneumonia. Eur Respir J.

[32] Ramírez P, Ferrer M, Martí V, Reyes S, Martínez R, Menéndez R (2011). Inflammatory biomarkers and prediction for intensive care unit
admission in severe community-acquired pneumonia. Crit Care Med.

[33] Kutz A, Briel M, Christ-Crain M, Stolz D, Bouadma L, Wolff M (2015). Prognostic value of procalcitonin in respiratory tract infections
across clinical settings. Crit Care.

[34] Park JH, Wee JH, Choi SP, Oh SH (2012). The value of procalcitonin level in community-acquired pneumonia
in the ED. Am J Emerg Med.

[35] Andrijevic I, Matijasevic J, Andrijevic L, Kovacevic T, Zaric B (2014). Interleukin-6 and procalcitonin as biomarkers in mortality
prediction of hospitalized patients with community acquired
pneumonia. Ann Thorac Med.

[36] Kasamatsu Y, Yamaguchi T, Kawaguchi T, Tanaka N, Oka H, Nakamura T (2012). Usefulness of a semi-quantitative procalcitonin test and the
A-DROP Japanese prognostic scale for predicting mortality among adults
hospitalized with community-acquired pneumonia. Respirology.

[37] Menéndez R, Cavalcanti M, Reyes S, Mensa J, Martinez R, Marcos MA (2008). Markers of treatment failure in hospitalised community acquired
pneumonia. Thorax.

[38] Shi Y, Xu YC, Rui X, Zhang HM, Wang Y, Du W (2014). Procalcitonin kinetics and nosocomial pneumonia in older
patients. Respir Care.

[39] Boussekey N, Leroy O, Alfandari S, Devos P, Georges H, Guery B (2006). Procalcitonin kinetics in the prognosis of severe
community-acquired pneumonia. Intensive Care Med.

[40] Rammaert B, Verdier N, Cavestri B, Nseir S (2009). Procalcitonin as a prognostic factor in severe acute exacerbation
of chronic obstructive pulmonary disease. Respirology.

[41] Bloos F, Marshall JC, Dellinger RP, Vincent JL, Gutierrez G, Rivers E (2011). Multinational, observational study of procalcitonin in ICU
patients with pneumonia requiring mechanical ventilation: a multicenter
observational study. Crit Care.

[42] Luyt CE, Guérin V, Combes A, Trouillet JL, Ayed SB, Bernard M (2005). Procalcitonin kinetics as a prognostic marker of
ventilator-associated pneumonia. Am J Respir Crit Care Med.

[43] Seligman R, Meisner M, Lisboa TC, Hertz FT, Filippin TB, Fachel JM (2006). Decreases in procalcitonin and C-reactive protein are strong
predictors of survival in ventilator-associated pneumonia. Crit Care.

[44] Hillas G, Vassilakopoulos T, Plantza P, Rasidakis A, Bakakos P (2010). C-reactive protein and procalcitonin as predictors of survival
and septic shock in ventilator-associated pneumonia. Eur Respir J.

[45] Boeck L, Eggimann P, Smyrnios N, Pargger H, Thakkar N, Siegemund M (2011). Midregional pro-atrial natriuretic peptide and procalcitonin
improve survival prediction in VAP. Eur Respir J.

[46] Tanriverdi H, Tor MM, Kart L, Altin R, Atalay F, SumbSümbüloglu V (2015). Prognostic value of serum procalcitonin and C-reactive protein
levels in critically ill patients who developed ventilator-associated
pneumonia. Ann Thorac Med.

[47] Dahaba AA, Elawady GA, Rehak PH, List WF (2002). Procalcitonin and proinflammatory cytokine clearance during
continuous venovenous haemofiltration in septic patients. Anaesth Intensive Care.

[48] Jensen JU, Hein L, Lundgren B, Bestle MH, Mohr TT, Andersen MH, Thornberg KJ, Løken J, Steensen M, Fox Z, Tousi H, Søe-Jensen P, Lauritsen AØ, Strange D, Petersen PL, Reiter N, Hestad S, Thormar K, Fjeldborg P, Larsen KM, Drenck NE, Ostergaard C, Kjær J, Grarup J, Lundgren JD, Procalcitonin and Survival Study Group (2011). Procalcitonin-guided interventions against infections to increase
early appropriate antibiotics and improve survival in the intensive care
unit: a randomized trial. Crit Care Med.

